# Is Mitochondrial Oxidative Stress a Viable Therapeutic Target in Preeclampsia?

**DOI:** 10.3390/antiox11020210

**Published:** 2022-01-22

**Authors:** Ramana Vaka, Evangeline Deer, Babbette LaMarca

**Affiliations:** 1 Center for Excellence in Cardiovascular and Renal Research, Department of Pharmacology, Physiology & Toxicology, University of Mississippi Medical Center, Jackson, MS 39216, USA; rvaka@ottawaheart.ca (R.V.); edeer@umc.edu (E.D.); 2 Center for Excellence in Cardiovascular and Renal Research, Department of Obstetrics and Gynecology, University of Mississippi Medical Center, Jackson, MS 39216, USA

**Keywords:** preeclampsia, mitochondrial oxidative stress, hypertension, antioxidants

## Abstract

Despite considerable research efforts over the past few decades, the pathology of preeclampsia (PE) remains poorly understood with no new FDA-approved treatments. There is a substantial amount of work being conducted by investigators around the world to identify targets to develop therapies for PE. Oxidative stress has been identified as one of the crucial players in pathogenesis of PE and has garnered a great deal of attention by several research groups including ours. While antioxidants have shown therapeutic benefit in preclinical models of PE, the clinical trials evaluating antioxidants (vitamin E and vitamin C) were found to be disappointing. Although the idea behind contribution of mitochondrial oxidative stress in PE is not new, recent years have seen an enormous interest in exploring mitochondrial oxidative stress as an important pathological mediator in PE. We and others using animals, cell models, and preeclamptic patient samples have shown the evidence for placental, renal, and endothelial cell mitochondrial oxidative stress, and its significance in PE. These studies offer promising results; however, the important and relevant question is can we translate these results into clinical efficacy in treating PE. Hence, the purpose of this review is to review the existing literature and offer our insights on the potential of mitochondrial antioxidants in treating PE.

## 1. Introduction

Preeclampsia (PE) is a pregnancy hypertensive disorder that affects approximately 5–7% of pregnancies worldwide [1,2]. PE leads to fetal growth restriction, morbidity, and mortality of both the mother and fetus with 70,000 maternal deaths and 500,000 fetal deaths reported annually [1]. Hypertension, endothelial dysfunction, inflammation, and oxidative stress are the hallmark characteristics of preeclamptic pathophysiology [2]. The importance of oxidative stress in preeclamptic pathology has been documented in preeclamptic patients as well as in animal models of PE. Multiple sources of oxidative stress have been identified in PE. Specifically, NADPH oxidases, mitochondrial dysfunction, nitric oxide synthase, and xanthine oxidases are known to be the major mediators [3]. In recent years, the phrase ‘mitochondrial oxidative stress in PE’ has attracted the attention of many research groups. PE placental mitochondria have been shown to exhibit increased oxidative stress markers and reduced levels of antioxidants [4,5,6]. Furthermore, studies using animal and cell culture models, have demonstrated the evidence for mitochondrial dysfunction and elevated mitochondrial reactive oxygen species (mtROS) production not only in the placenta but also in the endothelium and kidney [7,8,9]. Collectively, the large body of existing literature provides compelling evidence for mitochondrial oxidative stress in PE. While antioxidant therapies show a benefit in the animal models of PE, clinical trials on antioxidants (vitamins C and E) showed disappointing results [10,11]. Further, the DAPIT study (vitamins C and E for prevention of preeclampsia in women with type 1 diabetes) assessed the potential of antioxidants vitamins C and E in reducing preeclampsia risk in type 1 diabetic pregnant women [12]. While the study reported negative data on both primary (preeclampsia) and secondary outcomes (gestational hypertension and fetal outcomes), the subgroup analysis showed vitamin administration reduced PE incidence in women with low antioxidant levels at baseline in comparison to the placebo group. In addition to antioxidant vitamin trials, another drug that garnered substantial attention is aspirin. Low dose aspirin administration has been shown to attenuate placental oxidative stress in PE patients. The ASPRE (Aspirin for evidence-based PREeclampsia prevention) trial documented that administering aspirin (150 mg/day) from gestational week 11 to 36 reduces PE risk by 62% [13]. Nevertheless, none of these antioxidant clinical trials have resulted a meaningful benefit in treating PE. The possible reason behind these unsuccessful trials were thought to be due to the inability of the antioxidants to reach the site of oxidative stress (i.e., mitochondrial matrix). In support of this idea, we have recently shown targeting oxidative stress using mitochondrial specific antioxidants (MitoQ and MitoTempo) would attenuate hypertension and improve fetal outcomes in an animal model of PE [7]. Subsequent studies from ours and other laboratories identified a role of circulating pathogenic factors (i.e., soluble fms-like tyrosine kinase 1; sFlt1) and inflammatory cells or mediators (i.e., activated natural killer cells; NK cells, CD4+ T cells, tumor necrosis factor alpha; TNF-α; and agonistic autoantibodies for angiotensin II receptor type 1; AT1-AAs) in causing mitochondrial dysfunction in preclinical models of PE [14,15,16]. However, underlying molecular mechanisms are not well understood. Thus, further studies are vital for exploring these mechanisms to identify targets to combat mitochondrial dysfunction during PE. Drugs that can target existing ROS (antioxidants) may offer benefit in ROS-mediated oxidative damage, however, what may be of even greater benefit during PE is to identify therapeutic pathways that may target the inflammatory mediators upstream of mitochondrial dysfunction that have been shown to cause hypertension and IUGR. We outline this review beginning with general principles of mitochondrial function and ROS generation and a discussion of clinical and preclinical literature exploring mitochondrial dysfunction in preeclampsia, the use of antioxidants, and conclude with our insights on possible targets for the treatment for preeclampsia.

## 2. Mitochondrial Respiration and ROS Production

**Figure 1:**
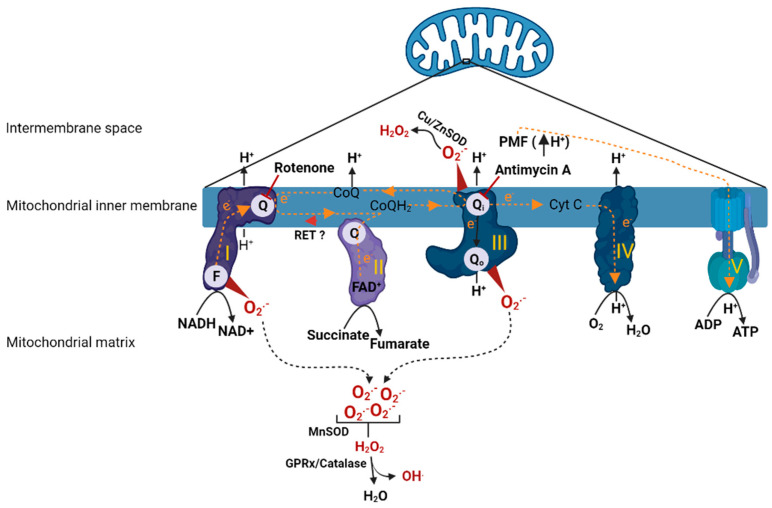
Animation depicting oxidative phosphorylation and ROS production in mitochondria. Once electrons have entered into the ETC, a series of redox reactions facilitate electron transfer within the ETC. Electron transfer leads to translocation protons from matrix to inner membrane space causing development of PMF, which drives Complex V to carry out phosphorylation of ADP. Direction of electron transfer or proton translocation are denoted with arrows. Complex I (FMN (or F) and quinone (or Q) binding sites) and Complex III (quinone (or Q) binding site) are the major sites of ROS production in the mitochondria. A concise depiction of ROS metabolism is also shown. Rotenone (Complex I inhibitor) and antimycin A (Complex III inhibitor) sites of action are indicated with inhibitory symbols. Reverse electron transfer from Complex II to Complex I is indicated with red arrowhead. ETC complexes are indicated by roman numerals. H^+^; proton, CoQ/CoQH_2_; coenzyme Q (oxidized/reduced), Cu/Zn SOD; copper/zinc superoxide dismutase, Cyt C; cytochrome C, e^−^; electron, F; FMN binding site, FAD^+^; flavin adenine dinucleotide (oxidized), FMN; flavin mononucleotide, GPRx; glutathione peroxidase, H_2_O; water, H_2_O_2_; hydrogen peroxide, MnSOD; manganese superoxide dismutase, NADH/NAD^+^; nicotinamide adenine dinucleotide (reduced/oxidized), O_2_; molecular oxygen, O_2_^●−^; superoxide, PMF; proton motive force, Q/Qi/Qo; CoQ binding sites, RET; reverse electron transfer. The figure was created using BioRender.

Mitochondria consists of two distinct membranes that surround the matrix; the outer membrane separates the inner membrane space from cytosol and is responsible for the mitochondrial motility in the cell. The inner membrane contains the electron transport chain (ETC), the main structure that performs respiration (oxygen consumption) (Figure 1). The ETC is composed of Complex I, II, III and IV, which as a single unit is coupled with Complex V (ATP synthase) to carry out oxidative phosphorylation (OXPHOS). During respiration, the reducing equivalents (NADH and FADH_2_) derived from metabolic processes (such as glycolysis and Krebs cycle) get oxidized by Complex I (NADH dehydrogenase) and Complex II (succinate dehydrogenase) which release a pair of electrons that travel through ETC by a series of redox reactions. The transfer of electrons through the complexes causes proton translocation from the matrix into the inner membrane space leading to the development of proton motive force (PMF) which in turn drives Complex V to phosphorylate ADP to form ATP. At Complex I, an electron pair received by flavin mononucleotide (FMN) in the matrix arm of Complex I is sent along to the ubiquinone binding site (Q binding site) on the membrane arm via 7 Fe-S clusters. The electrons reached at the Q binding site are utilized to reduce electron carrier, ubiquinone (CoQ) to ubiquinol (CoQH_2_) and the reduced CoQH_2_ binds to the quinone binding site on the Complex III (Qi/Qo) where it releases the electron pair by oxidation. Further, the electron pair from Complex III are carried by another electron carrier, cytochrome c (Cyt C), to Complex IV where they are utilized to reduce molecular oxygen (O_2_). Similarly, at Complex II, electrons received from oxidation of FADH_2_ are sent to Q binding site via Fe-S clusters and are utilized to reduce CoQ to CoQH_2_ (ubiquinol). The electrons in COQH_2_ go to Complex III and then to Complex IV by similar redox reactions as described in the case of Complex I [17].

Mitochondria under normal conditions produce small amounts of ROS that participate in physiological functions such as regulating vascular tone, sensing oxygen tension, membrane receptor signal transduction, and redox homeostasis [18]. However, the dysfunctional mitochondria produce excess amounts of ROS, several fold higher than levels that are seen in physiological conditions [19]. This is thought to be due to impaired ETC complexes and their compromised ability to carry out electron transfer. Complex I and III are the major ROS-generating sites within the mitochondria [19]. Specifically, the mechanisms by which Complex I produces ROS are via (1) forward electron transport [20], (2) or reverse electron transport [21]. In the first case, conditions such as low ATP demand or loss of Cyt C or damage to any of the complexes would lead to buildup of electrons at the FMN site, where O_2_ reacts with the reduced form of FMN causing ROS production. This is the mechanism by which rotenone, a Complex I inhibitor, (by binding to Q binding site) produces excessive ROS. Another mechanism by which Complex I causes ROS production is via reverse electron transport (RET), which occurs due to back travel of electrons from Complex II to Complex I. RET is favored in the presence of highly reduced levels of CoQ or high ΔP (PMF). These situations force electrons back from CoQH_2_ into Complex I, and reduction of FMN and NAD+ causes ROS production. Another important site of ROS generation within the ETC is Complex III [22,23]. Complex III mediates transfer of electrons from reduced Q pool (from Complex I/II) to Cyt C. Complex III has two distinct sites, Qi and Qo, where the quinol (CoQH_2_) undergoes oxidation to deliver electrons to Cyt C. When a Qi site is inhibited with antimycin A, the Qo site generates large amounts of superoxide (O_2_^●−^) due to stable semiquinone form (intermediate form) within the Qo site. However, when Qi is not inhibited with antimycin A or with no defects in Complex III otherwise, superoxide production from Complex III is insignificant. At Complex III, the superoxide is produced in both sides of inner membrane (matrix and inner membrane space). The superoxide produced in the inner membrane space subsequently is dismuted to H_2_O_2_ by superoxide dismutase (Cu/Zn SOD), which will eventually diffuse into the cytoplasm. In addition to the Complex I and III, intramitochondrial enzymes such as α-glycerophosphate dehydrogenase, α-ketoglutarate dehydrogenase, and dihydroorotate dehydrogenase are known to produce ROS although their contribution is relatively low in comparison to Complex I and III [22].

The excessive mtROS production can cause membrane lipid peroxidation, damage the respiratory chain, and release of Cyt C, leading to initiation of apoptotic pathways ultimately causing cell death [24,25]. During increased ROS generation, the antioxidant systems within the mitochondria counteract the effects of these reactive oxidant molecules. For example, mitochondrial superoxide dismutase (MnSOD) catalyzes the redox reaction of O_2_^●−^ into O_2_ and H_2_O_2_. The produced H_2_O_2_ is quickly fully reduced to H_2_O and O_2_. Further, glutathione peroxidase hydrolyzes H_2_O_2_ to H_2_O by a coupled reaction involving oxidation of glutathione. H_2_O_2_ can react with ferrous ions, which in turn may be partially reduced to highly reactive hydroxyl radical hydroxyl radicals (OH^.^). Additionally, superoxide can react with nitric oxide (NO) to produce highly reactive peroxynitrate. However, excessive production of ROS or disruption to the antioxidant defenses leads to extensive oxidative damage. Overall, the fine balance between ROS production and endogenous antioxidant reserves determines the extent of oxidative stress within a given local environment.

## 3. Mitochondrial Dysfunction in Preeclampsia

### 3.1. Clinical Studies

The first report that a family with mitochondrial (mt) dysfunction had a high incidence of preeclampsia/eclampsia was published three decades ago [26]. Since then, a huge amount of literature has centered on the role of mitochondrial function or dysfunction in PE has accumulated. The studies published shortly after this first report showed evidence of abnormal mt morphology, oxidative stress, and reduced Complex IV activity in PE placental sections [27,28,29]. Further, a recently published study from Zsengeller et al. confirmed reduced Complex IV activity in the isolated mitochondrial membranes from PE placenta [30]. Moreover, Muralimanoharan et al. documented placental mitochondrial fraction from PE women showed reduced Complex III activity with low expression of Complexes I and IV [31]. In line with these reports, our recently published study was one of the first to compare bioenergetics of placental mitochondria of term (GA > 34 weeks) and preterm (GA < 34 weeks) PE patients [32]. In this work, we documented term PE placental mitochondria in the presence of glutamate, malate, and ADP (Complex I mediated state 3), respire at a significantly low rate in comparison to normal pregnant placental mitochondria. Further, either preterm or term PE placental mitochondria exhibit low maximal respiration rates (induced in the presence of an uncoupler; FCCP) in comparison to normal pregnant mitochondria. Similarly, in the presence of succinate and ADP, both term and preterm placental mitochondria showed a significantly low respiration rate in comparison to normal pregnant mitochondria. Moreover, while preterm PE placental mitochondria showed significant reductions in maximal respiration rate, term PE placenta showed a trend towards reduction. Furthermore, we found that both expression and activity of Complex IV were significantly reduced in term PE placental mitochondria. Interestingly, we did not find changes in expression of other complexes, albeit reduced Complex IV could explain reduced respiration rates seen in term PE placental mitochondria. However, two independent studies by other research groups revealed that preeclamptic placental state 3 (preterm PE; GA-30 weeks) or maximal respiration (term PE; GA-37 weeks) rates were significantly increased in comparison to control normal pregnancies [33,34]. Furthermore, while term PE placenta exhibits increased Complexes II and III expressions, no changes were seen in any of the complex levels in preterm PE placenta. In contrary to these reports documenting altered mitochondrial respiration rates in PE, Mando et al. reported no changes in respiration rates in primary trophoblasts isolated from PE placenta in comparison to normal pregnancies [35]. It is worth noting that in their study, respiration measurements were collected in cytotrophoblasts isolated from placental tissue. Thus, these findings do not completely represent a whole placental mitochondria biogenetic profile, unlike studies that documented altered respiration rates in which mitochondria were prepared from whole placental tissue.

Holland et al. reported preterm PE placental mitochondria exhibit low H_2_O_2_ production in comparison to control pregnancies, and further showed that SOD expression in PE placental mitochondria was significantly low [34]. Our study also assessed real-time mtROS production in isolated mitochondria. Interestingly, both term and preterm PE mitochondria showed significantly low mtROS production in comparison to normal pregnant mitochondria [32]. This is particularly an interesting finding as most often reduced respiration rates are associated with increased ROS generation. This suggests that low H_2_O_2_ levels seen in preeclamptic placentas may be a result of low expression of ROS metabolic systems. The excessive ROS produced within the mitochondria can cause lipid peroxidation, ETC damage, mtDNA release, ultimately apoptosis and cell death [23]. In fact, a few earlier studies reported increased amounts of lipid peroxides, isoprostanes, protein carbonylation, and malondialdehyde (MDA) levels in preeclamptic placenta [4,36,37]. It is important, however, to note that oxidative stress is not only a result of increased pro-oxidants but also can result from reduced antioxidant levels altering pro vs. anti-oxidant balance. The expression and activity of antioxidant systems MnSOD and glutathione/glutathione peroxidase were reported to be reduced in preeclamptic placenta [36,38]. However, a few other studies showed increased levels of thiol/disulfide oxidoreductases, glutathione levels, glutathione peroxidase, and peroxiredoxin III/SP-22 in preeclamptic placenta [39]. The possible explanation for this increase in antioxidants could be a positive feedback induction of antioxidants to overcome the excessive oxidative stress resulting from mtROS production. Further, another consequence of mt dysfunction is the release of mtDNA from the damaged cells into the local milieu and systemic circulation. A few studies reported elevated levels of mtDNA in both placenta and blood samples of PE patients [8,40,41,42]. The mtDNA can act as a damage-associated molecular pattern (DAMP) and elicit immune response via activation of TLR-9 signaling and TNF-α production. In hopes of dissecting molecular mechanisms underlying mitochondrial damage in PE placenta, recent studies are exploring mechanisms beyond bioenergetics. For example, a recently published study by Zhou et al. revealed dysregulated BNIP3 (Bcl-2/adenovirus E1B 19-kDa interacting protein 3; protein involved in mitophagy) was shown to mediate mitochondrial damage and apoptosis in preeclamptic placenta, suggesting a role for impaired mitophagy regulation in preeclamptic pathophysiology [43].

### 3.2. Animal Studies

Following early clinical observations of evidence for mitochondrial dysfunction in PE, several research groups set out to explore mitochondrial oxidative stress in animal models of PE. We were the first to establish a functional link between hypertension and mitochondrial dysfunction in PE. In our published study utilizing a RUPP (reduced uterine perfusion pressure) rat model, we demonstrated that preeclamptic rats exhibit reduced mitochondrial respiration in isolated mitochondria from placenta or kidney [7]. Interestingly, this reduced respiration was associated with elevated ROS production in both placental and renal mitochondria. Assessing electron transport chain activity further revealed that Complexes I, II, and IV were impaired in mitochondria of preeclamptic rats. Subsequently, our preclinical studies exploring the mechanistic contribution of mtROS in PE consistently reproduced these novel findings [15,16]. Similarly, a recent study by Yang et al. showed that RUPP mice exhibit increased placental mitochondrial oxidative stress along with mitochondrial damage [44]. Moreover, because the kidney is an important regulator of blood pressure and one of the affected organs in PE patients, we expanded our exploration to renal mitochondria. As we suspected, PE rats displayed impaired mitochondrial function and increased ROS production in isolated renal mitochondria. These findings were confirmed by a recently published study that documented increased mtROS production in kidneys from RUPP preeclamptic rats [8]. These exciting findings prompted research groups to explore mitochondrial bioenergetics in organ systems beyond placenta or kidneys. For instance, a recent study published by Booz et al. demonstrated that isolated cardiac mitochondria from RUPP rats show reduced mitochondrial respiration and Complex IV activity [45]. Further, Sánchez-Aranguren et al. reported that overexpression of sFlt1 in heme oxygenase-1-deficient mice causes mitochondrial dysfunction in cardiac mitochondria [46]. Collectively, studies reporting mitochondrial dysfunction in extra placental organ systems suggest a role for circulating factors released in response to placental ischemia mediating maternal mitochondrial damage. While these studies collectively document an evidence for excessive mitochondrial oxidative stress in a RUPP rodent model of PE, studies exploring mitochondrial dysfunction in other animal models of PE have also emerged in the recent years. Utilizing an ischemia/reperfusion pregnant rat model of PE, Okatani et al. showed reduced mitochondrial function and increased TBARS (marker of elevated oxidative stress) in the placenta of PE rats [47]. Further, a study by Chen et al. documented reduced Complex IV expression and increased oxidative stress markers in the placenta of a HIF-1α-induced PE mouse model [48]. Furthermore, utilizing a NaCl rat model of PE, Popova et al. demonstrated administering 1.8% NaCl to the pregnant rats increases state 4 respiration and reduces respiratory control ratio in the placental isolated mitochondria [49].

### 3.3. In Vitro Cell-Based Studies

Literature on exploring the mitochondrial role in PE pathology also encompasses mechanistic exploration studies utilizing cell-based assays. While trophoblast cell lines were used to assess placental mitochondrial function, vascular endothelial cell lines were used to understand the effect of placental ischemia-induced circulating factors on mitochondrial dysfunction in the maternal systemic vasculature. Sánchez-Aranguren et al. showed that HTR-8/SVneo cells (trophoblast cell line) treated with 2% PE patient serum exhibit reduced mitochondrial respiration and elevated mtROS production [14]. We showed that HUVECs (human umbilical vein endothelial cells) treated with 10% RUPP rat serum caused increased mtROS production [7]. In line with our finding, McCarthy et al. reported that HUVECs treated with 3% plasma from PE patients not only increased mtROS production but also reduced cellular mitochondrial respiration [9]. Further, Sánchez-Aranguren et al. demonstrated that treating either HUVECs or HTR-8/SVneo cells with 2% PE patient serum reduced cellular respiration, and increased mtROS production [14]. Moreover, our recently published study confirmed that PE patient serum causes impaired mitochondrial function and excessive mtROS generation in HUVECs [50]. Taken together, these studies suggest that circulating factors released in response to placental ischemia in PE placenta leads to mitochondrial dysfunction and ROS production in both placental and vascular cells. Specifically, in regard to our findings, although real-time placental mtROS was not elevated in our patient cohort, we demonstrate that soluble factors from PE women stimulate mt ROS in the vascular endothelium, thus further supporting the role for peripheral mtROS in patients with PE.

## 4. Mediators of Mitochondrial Dysfunction and Excessive ROS Production in Preeclampsia

**Figure 2:**
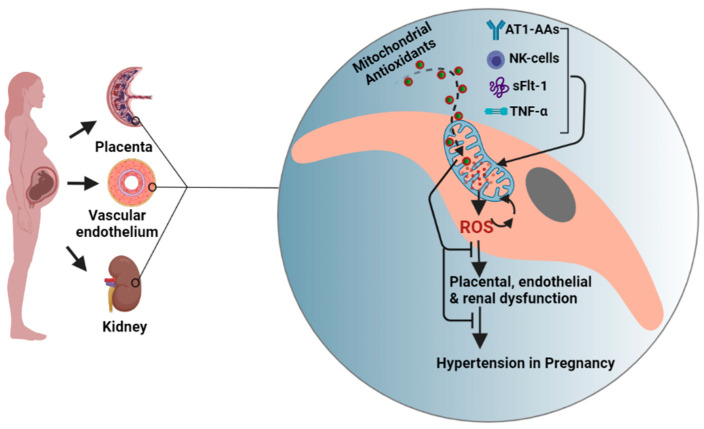
Conceptual figure showing organ systems and the mechanisms and the events leading to hypertension in preeclampsia. We here show a hypothetical cell from placenta orvascular endothelium orkidney to indicate mtROS mechanisms and targeted antioxidants strategy. We show TNF-α, sFlt1, AT1-AAs, and activated NK cells are some of the important pathological factors driving mitochondrial ROS in preeclampsia. Mitochondrial antioxidants MitoQ and MitoTempo accumulate in the cytoplasm (10-fold) and mitochondrial matrix (100–500-fold) based on membrane potential leading to abrogation of mitochondrial oxidative stress and organ dysfunction and hypertension. TNF-α, tumor necrosis factor; AT1-AA, agonistic antibodies to the angiotensin II receptor type 1; NK cell, natural killer cell; ROS, reactive oxygen species; sFlt1, soluble fms-like tyrosine kinase-1. The figure was created using BioRender.

The studies exploring pathological mediators and molecular mechanisms that underlie mitochondrial dysfunction and oxidative stress are still at their inception. Our work over the past few years has identified inflammatory cells or mediators; activated NK cells, CD4+ T cells, AT1-AAs, and TNF-α as some of the crucial players in mediating mitochondrial oxidative stress in PE (Figure 2). We have shown that administration of AT1-AAs (GD13–19) to normal pregnant rats causes elevated mtROS production in placenta and kidney [51]. Further, these findings were confirmed in our subsequent study, in which we reported inhibition of AT1-AAs by coadministration with 7 amino acid peptide (n7AAc) reduces mtROS in preeclamptic rats [15]. Importantly, we have shown that in vitro treatment of endothelial cells exposed to 10% PE patient serum with n7AAc abrogates mtROS production and improves cell respiration [50]. We suspect that AT1-AA-mediated mitochondrial oxidative stress involves multiple downstream pathways. For instance, Angiotensin II (Ang-II) has been shown to cause mitochondrial dysfunction via cross talk with NADPH oxidase [52]. Since AT1-AAs signal through the AT1 receptor similarly to Ang-II, it would be reasonable to speculate that AT1-AAs would mediate mitochondrial dysfunction via activation of NADPH oxidase. Another downstream pathway we have identified was NK cell activation. Granzymes A and B, serine proteases majorly secreted by activated NK cells, have been shown to cause mitochondrial dysfunction and mtROS in in vitro studies as well as in isolated mitochondria [53,54]. Further, the mechanisms underlying granzyme-mediated mitochondrial oxidative stress are shown to be mediated via cleavage of Complex I subunits [55] and caspase substrates such as BID [56]. In support of these findings, we showed that depletion of NK cells in PE rats improves mitochondrial function [16]. Further, we previously demonstrated that AT1-AAs activate NK cells in pregnant rats [51]. Hence, it is possible that NK cell-induced mitochondrial oxidative stress is downstream of AT1-AA signaling via the AT1 receptor. Moreover, based on the existing literature on the ability of TNF-α to cause mitochondrial oxidative stress or hypertension, and reported moderately high levels of TNF-α in PE, we hypothesized that TNF-α causes mitochondrial oxidative stress in pregnant rats. When we infused TNF-α into normal pregnant rats, we noted increased mitochondrial oxidative stress along with a preeclamptic phenotype [57]. Further, in a separate study, we showed that administering etanercept (TNF-α inhibitor) to PE rats (single dose of 0.4 mg/kg, s.c, on GD18) reduces mtROS in both placental and renal mitochondria [58]. Further, we have previously shown TNF-α infusion in the pregnant rats increases circulating AT1-AA levels [59]. Thus, it is possible that TNF-α-mediated mitochondrial oxidative stress is both upstream and downstream of AT1-AA signaling through the AT1R; however, future studies blocking the activity of the AT1-AA in response to TNF-α are needed to answer this question. Further, we have recently shown the adoptive transfer of CD4+ T cells into normal pregnant rats causes mtROS and mitochondrial dysfunction, which was prevented by administration of Orencia, revealing a novel role of CD4+T cells in causing mitochondrial oxidative stress in response to placental ischemia [60]. Subsequent studies from our lab showed interleukin-2 (IL-2) prevents mitochondrial ROS and dysfunction in PE rats, which may be through changes in CD4+T cell or NK cell populations [61]. In addition, soluble fms-like tyrosine kinase 1 (sFlt1), an antiangiogenic factor shown to mediate preeclamptic pathology, has also been shown to play a role in causing mt ROS in PE [14]. We followed these studies and demonstrated sFlt-1 stimulates placental and endothelial mitochondrial ROS and a role of progesterone- induced blocking factor (PIBF) to play a defensive role against hypertension and placental and endothelial mitochondrial function during pregnancy [62].

Jiang et al. showed that injecting pregnant mice (GD9–17) with sFlt1 resulted in swollen mitochondria in trophoblast cells with increased oxidative stress and reduced antioxidant markers [63]. Further, a study published by Brownfoot et al. reported that metformin, a known mitochondrial Complex I inhibitor, reduced the secretion of sFlt1 and soluble endoglin (sEng) from primary endothelial cells, villous cytotrophoblast cells, and preterm preeclamptic placental villous explants [64]. In a separate study, this group of investigators reported that treating cytotrophoblasts with rotenone (Complex I inhibitor), antimycin A (Complex III inhibitor), and oligomycin (Complex V inhibitor) significantly reduced sFlt1 secretion, and treatment with metformin or esomeprazole significantly reduced mitochondrial respiration in the trophoblasts [65]. These studies were the first to show sFlt1 production is regulated by ETC. Commensurate with these findings, Sánchez-Aranguren et al. showed that endothelial cells and trophoblasts treated with sFlt1 (50 ng/mL) show significant reduction in maximal respiration rate, and interestingly, sFlt1 treatment switched metabolism to glycolysis in endothelial cells, while this was not observed in trophoblasts. Furthermore, reduced respiration was associated with increased mtROS production and reduced mitochondrial membrane potential in endothelial cells but not in trophoblasts [14]. Authors explained the differential actions of sFlt1 by suggesting that trophoblast cells, in early stages of gestation, are metabolically programmed to overlap and compensate for the effects of metabolic disruptors. Overall, this study demonstrated an important role of sFlt1 in mitochondrial dysfunction and metabolic reprogramming in endothelial cells. Further, another study reported an inverse correlation between expression of sFlt1 and mitochondrial complex IV activity in preeclamptic placentas [30]. One important observation, however, is that studies by Brownfoot et al. and Hastie et al. show that sFlt1 production is reduced when ETC is inhibited, implying that reduced ETC activity should in fact lower sFlt1 production. However, preeclampsia is associated with reduced ETC activity and increased sFlt1 production. Hence, further studies exploring ETC-regulated production of sFlt1 are necessary to better understand sFlt1 regulation of mitochondrial dysfunction in PE. Collectively, studies from Brownfoot et al., Hastie et al., and Sánchez-Aranguren et al. indicate that Complex I regulates sFlt1 production and its signaling within the mitochondria. We have shown that infusion of AT1-AAs into pregnant rats or mice results in elevated circulating sFlt1 levels [66], suggesting sFlt1 production is also downstream of AT1-AA signaling. Overall, these studies collectively identify Complex I as a possible source of mtROS in PE mitochondria. Reverse electron transfer (RET) is an important ROS production mechanism at Complex I. Importantly, all our mtROS data were generated in isolated mitochondria using Complex II substrate (succinate) in the absence of Complex I substrates [7,15,16]. This supports a hypothesis of possible reverse electron transfer of electrons to Complex I from Complex II leading to mtROS at Complex I. However, in our PE rat mitochondria (although not in PE patient), Complex I activity is significantly impaired, suggesting that reverse transfer of electrons coming from Complex II to I would not have occurred, indicating that reverse flow of electrons to FMN site is not feasible in these mitochondria. However, incorporation of rotenone (to block RET, if any) in our assay would confirm if RET in fact was one of the mechanisms of ROS production in PE rat mitochondria. Additionally, mtROS measurements in the presence of Complex I substrates (glutamate/malate) and rotenone without Complex II substrate (succinate) would reveal the contribution of ROS during forward flow at Complex I. Complex III is another well-known site of ROS production in the mitochondria. Muralimanoharan et al. documented that Complex III activity is reduced in PE placental mitochondria [31]. However, studies focused on Complex III are limited to date. Thus, mtROS measurements in isolated mitochondria using a combination of Complex I or II substrates along with rotenone and antimycin A would reveal the relative contributions of Complex I and III in generating ROS in the mitochondria. An important to consideration to keep in mind is that both Complex I and II substrates are available in the cells and, depending on the intact activities of complexes, both forward and reverse flow electron transport could simultaneously contribute to ROS generation at Complex I and Complex III. Hence, one should be careful in drawing conclusions from the experiments conducted using isolated mitochondria. Lastly, no studies thus far have been conducted to examine the contribution of other known ROS generating sources such as α-glycerophosphate dehydrogenase, α-ketoglutarate dehydrogenase, and dihydroorotate dehydrogenase, and hence further studies are warranted for a thorough understanding of mtROS production in PE.

In summary, the existing literature suggests that manifestation of mitochondrial oxidative stress in PE involves a complex interplay of AT1-AAs, activated NK cells, TNF-α, and sFlt1.

## 5. Mitochondrial Targeted Antioxidants in Preeclampsia

Although it has been four decades since the early findings on the pathological role of mitochondrial dysfunction in PE patients emerged, it was not until recently the investigators started embracing the potential of mitochondrial antioxidants to treat PE. The studies assessing mitochondrial antioxidants in preeclamptic models are summarized in Table 1. The impetus to explore mitochondrial targeted antioxidants in PE in part stems from the literature on mitochondrial targeted antioxidants to treat hypertension in preclinical models. Dikalova et al. showed infusion of malate or mitoebselene (mitochondrial targeted H_2_O_2_ scavenger)-attenuated Ang-II induced hypertension in mice. Further, administration of mitochondria-targeted SOD mimetic (MitoTempo) significantly attenuated Ang-II-induced hypertension in mice [52]. Furthermore, Graham et al. reported that 8-week treatment of spontaneously hypertensive rats with mitochondria-targeted antioxidant (MitoQ) significantly reduced systolic blood pressure by 25 mmHg [67]. Based on this literature, a few research groups in recent years, including ours, have begun to explore mitochondrial-targeted antioxidants in in vitro and in vivo preclinical models of PE.

Antioxidant molecules can chemically be linked with the lipophilic cation triphenyl phosphonium (TPP) to enable selective accumulation of antioxidants into the mitochondrial matrix based on membrane potential [68]. MitoQ, first in class (developed by Michael Murphy’s group at Medical Research Council, Cambridge, UK), was evaluated in several animal disease models, and clinical trials of Parkinson’s disease and liver disease. Soon after, other TPP-tagged antioxidative molecules such as MitoTempo and Mito Vitamin E were developed. The exploration of mitochondrial antioxidants in PE is relatively new, and MitoQ and MitoTempo are the only two mitochondrial targeted antioxidants that have been assessed in preeclamptic animal models thus far.

### 5.1. MitoQ

Coenzyme Q 10 (also known as ubiquinone or CoQ) is a lipophilic molecule bearing a fully substituted benzoquinone ring, and a hydrophobic poly-isoprenoid tail is located within the inner mitochondrial membrane [68]. The main role of CoQ is to shuttle electrons from Complex I or Complex II to Complex III [68]. CoQ in its semiquinone form (during partial reduction in Q cycle at the Complex III) produces O_2_^●−^, however, when it is in a fully reduced state it acts as an antioxidant [69]. PE has been shown to be associated with reduced levels of CoQ [70]. Xu et al. showed that administration of CoQ10 to L-NAME-induced preeclamptic rats resulted in significant lowering of blood pressure and proteinuria with improved fetal outcomes along with increased mtDNA and mitochondrial membrane potential [71]. Despite seeing therapeutic benefit with CoQ in animal models of disease, higher doses of CoQ may need to be considered in clinical trials to ensure its therapeutic effectiveness [72]. Large doses of CoQ are thought to be necessary due to insufficient CoQ accumulation at the intended site of action (mitochondrial matrix). To overcome this challenge, Smith et al. developed Mito Q by joining CoQ to the lipophilic cation triphenylphosphonium (TPP) [73]. Soon thereafter, several studies characterized and evaluated MitoQ in in vitro and in vivo disease models. MitoQ accumulates 10-fold higher in the cytoplasm and 100–500-fold higher in the mitochondrial matrix [73]. Once accumulated, MitoQ is adsorbed on to the matrix surface of the inner membrane, where it is reduced to its active quinol form (antioxidant) by Complex II. During its antioxidant reactions with radicals, it is oxidized to quinone form, which will be re-reduced by Complex II to quinol, restoring the antioxidant activity. However, Mito Q cannot participate in electron transfer like the natural CoQ as it is poorly oxidized by Complex III [74]. Thus, its beneficial effects are solely attributed to its antioxidant ability. Although its antioxidant effects are thought to be mediated by its ability to lower lipid peroxidation, other mechanisms such as detoxification of superoxide and peroxynitrate have also been proposed [70]. Several animal studies demonstrated benefits of MitoQ in a wide range of disease models such as Parkinson’s disease, kidney, liver, and heart disease [75,76,77,78]. These animal studies were translated into clinical studies on Parkinson’s disease (www.clinicaltrials.gov, NCT00329056, accessed on 20 December 2021), chronic hepatitis C virus (www.clinicaltrials.gov, NCT00433108, accessed on 20 December 2021), diastolic dysfunction (www.clinicaltrials.gov, NCT03586414, accessed on 20 December 2021), and multiple sclerosis (www.clinicaltrials.gov, NCT04267926, accessed on 20 December 2021).

We have recently demonstrated that MitoQ reduced hypertension and improved fetal outcomes in preeclamptic rats [7]. In line with our findings, Yang et al. have recently showed administering MitoQ during late gestation to preeclamptic mice attenuated hypertension and kidney damage [44]. However, interestingly, they showed that administering MitoQ during early gestation exacerbated hypertension, fetal growth restriction, and proteinuria. The authors explained these findings by suggesting MitoQ prevents mild oxidative stress in the early gestation, which is essential for normal placentation during early gestation, and hence attenuating mild oxidative stress resulted in unwanted effects. Based on these interesting findings, we believe that the benefits of MitoQ in preeclamptic rats were possibly due to reducing oxidative stress during late gestation, since we administered MitoQ during late gestation (GD14–19). However, further studies focusing on dynamics of oxidative stress regulation during the entirety of gestation would further advance our knowledge on the pathophysiological role of oxidative stress in pregnancy. Further, from a mechanistic point of view, we hypothesized that attenuation of hypertension partly resulted from reversal of endothelial dysfunction. We have shown that endothelial cells treated with serum obtained from MitoQ treated preeclamptic rats completely reversed mtROS when compared to cells exposed to sera from untreated PE rats, supporting the theory that circulating factors play an important role in causing endothelial mitochondrial oxidative stress and dysfunction [7]. Additionally, MitoQ-treated preeclamptic rats showed significant improvement in respiration and ETC complex activities (Complex I and IV). Moreover, these data imply that the improved fetal outcomes observed in this study are due to improved mitochondrial function locally within the placental cell types. However, a critical consideration to keep in mind with the drugs administered to pregnant mothers is to ensure no passage across the placenta and accumulation in the fetus. Addressing this challenge, Philips et al. evaluated if tagging MitoQ to nanoparticles (NP) would keep it from crossing the placenta and affecting fetal development. Their study revealed that a single dose (0.5 µM) of NP-bound MitoQ prevented placental oxidative stress, improved fetal weights, and protected fetal brain in the hypoxic pregnancy rat model (11% oxygen: GD16–21) [79]. As expected, MitoQ was not detected in any of the fetal tissues examined (brain and liver), yet fetal benefits seen in this study are believed to be due to MitoQ actions in the placenta rather than on the fetus. However, incorporating additional study group with MitoQ untagged to NP would have served as a good control group in this study.

**Table 1:** Studies assessing mitochondrial antioxidants in preeclamptic models.

Antioxidant	Model		Treatment			Findings	Ref.
	In Vitro	In Vivo	Dose	Duration	Route		
**MitoQ**		L-NAME rat model ^a^	60 mg/kg/day	5 days (GD 15–20)	Oral	Lower blood pressure Lower proteinuria Improve fetal outcomes Reduce mtROS/oxidative stress Improve mt function	[71]
		RUPP rat model	500 µM/kg/day	5 days (GD 14–19)	Oral	Lower blood pressure Improve fetal outcomes Improve mt function	[7]
		RUPP mouse model ^b^	100 µM/kg/day	4 days (GD 13.5–17.5)	Oral	Lower blood pressure Improve fetal outcomes Lower proteinuria Improve renal function	[43]
		Hypoxia rat model	125 µM	5 days (GD 15–20)	I.V	Improve fetal outcomes	[78]
		Hypoxia rat model	500 µM/kg/day	14 days (GD6–20)	Oral	Improve fetal outcomes Reduce mt oxidative stress	[79]
	RUPP serum- treated HUVECs		10% serum ^c^	Overnight	Direct addition	Reduce mt oxidative stress	[7]
	H_2_O_2_ treated HTR8-S/Vneo trophoblast cells		1 µM		Direct addition	Reduce oxidative stress	[43]
**MitoTempo**		RUPP rat model	1 mg/kg/day	5 days (GD 14–19)	Oral	Lower blood pressure Improve fetal outcomes Improve mt function	[7]
	PE serum treated HUVECs		5 µM	2 h	Direct addition	Reduce mt oxidative stress	
	H_2_O_2_ treated HTR8-S/Vneo trophoblast cells		1 µM		Direct addition	Reduce oxidative stress	[43]
**Ergothioneine**		RUPP rat model	25 mg/kg/day	8 days (GD11–19)	Oral	Lower blood pressure Improve fetal outcomes Reduce mt oxidative stress	[8]
		RUPP rat model	25 mg/kg/day	8 days (GD11–19)	Oral	Reduce mt oxidative stress	[19]

GD: gestational day; H_2_O_2_: hydrogen peroxide; HUVECs: human umbilical vein endothelial cells; I.V: intravenous; RUPP: reduced uterine perfusion pressure; mt: mitochondria; L-NAME: N^ω^-Nitro-L-arginine methyl ester; PE: preeclampsia. ^a^ Study utilized untagged CoQ10. ^b^ MitoQ or MitoTempo administration during late gestation attenuates PE symptoms while administration during early gestation exacerbates PE-like phenotype. ^c^ Treated with 10% serum collected from MitoQ-administered rats.

Furthermore, another study reported that pregnant rats exposed to hypoxia (13–14% oxygen; GD6–20) showed increased mitochondrial stress in placenta, as seen by increased upregulation of glucose-regulated protein 78 (GRP-78) and activating transcription factor 4 (ATF-4); however, MitoQ treatment (500 μmol/L in drinking water; GD6–20) prevented mitochondrial stress. Interestingly, MitoQ uptake by the placenta and maternal liver was considerably greater than that of the fetal liver, suggesting that fetal improvements from MitoQ supplementation is via actions directly on the placenta [80]. This study further supports that mitochondrial-targeted antioxidants are beneficial in complicated pregnancies with a hypoxic placental environment. However, this study highlights the question of whether there is any need for subjecting MitoQ to formulation technology (such as nanoparticles) to limit its fetal accumulation. Considering the data from only a few studies on MitoQ administration in pregnancy, it may be too early to conclude that MitoQ does or does not cross the placenta. Further studies focusing on MitoQ disposition in the feto–placental unit using in vitro/ex vivo models would provide us a better understanding of MitoQ pharmacokinetics in pregnancy.

### 5.2. MitoTempo

MitoTempo is a piperidine nitroxide radical Tempol (4-hydroxy-2,2,6,6,-tetramethyl-piperidine-1-oxyl) conjugated to TPP. Tempol is an SOD mimetic that dismutes superoxide, thereby preventing its conversion to peroxynitrate, a highly reactive damaging oxidant molecule. Tempol has been proven to be effective in not only preventing onset of preeclampsia, but also in reversing the disease in animal models of PE. Our group has previously shown that treating RUPP rats with Tempol (at dose of 1 mg/kg/day for 4 days, GD14–19) reduced blood pressure to the level of normal pregnant rats [81]. Further, we reported that hypertension caused by administration of IL-17 [82] or AT1-AAs [83] in normal pregnant rats was reversed by Tempol. In line with our studies, Hoffman et al. showed that administering Tempol to spontaneous preeclamptic mice (BPH/5) reduces hypertension and proteinuria, and improves placental–fetal outcomes [84]. MitoTempo has been shown to be effective in attenuating hypertension in Ang-II or DOCA-salt-sensitive models of hypertension [52]. Interestingly, in the same study, non-targeted Tempol at the same dose level used as a control did not attenuate hypertension in Ang-II-infused mice. However, previously published studies showed a therapeutic benefit of non-targeted Tempol in hypertension models, but Tempol doses used in those studies were high (~1000 fold) in comparison to the MitoTempo or Tempol dose in the Ang-II study. This suggests, as with other non-targeted antioxidants, high doses of Tempol are required to achieve adequate concentrations at the site of ROS production (mitochondrial matrix).

We have recently reported RUPP rats treated with MitoTempo (at a dose of 1 mg/kg per day, GD 14–19) show significant attenuation in elevated blood pressure [7]. Further, as seen with MitoQ, endothelial cells treated with sera from MitoTempo-treated PE rats showed significantly low levels of mtROS production, indicating the potential of MitoTempo in protecting endothelial cells from circulating factors. Commensurate with our findings, McCarthy et al. showed that endothelial cells (HUVECs) pretreated with MitoTempo (5 μM) for 2 hours prior to incubation with 3% plasma from PE patients resulted in significant reduction in mtROS production [9]. As seen in the models of hypertension, non-targeted Tempol showed benefits at a dose of 30 mg/kg/day in PE models; however, in our in vivo MitoTempo study, we showed a therapeutic benefit at a dose of 1 mg/kg/day, which was 30-fold lower than the non-targeted Tempol dose, indicating the importance of using mitochondrial-targeted Tempol in PE.

Thus far, no clinical studies have evaluated MitoTempo, however, untagged Tempol is being evaluated in prostate cancer (www.clinicaltrials.gov, NCT04337099, accessed on 20 December 2021), and in toxicities associated with cisplatin and radiation treatment in head and cancer patients (www.clinicaltrials.gov, NCT03480971, accessed on 20 December 2021).

### 5.3. Ergothioneine

Ergothioneine (ERG) is a water-soluble amino acid derived from dietary sources usually synthesized by fungi and bacteria [85]. The antioxidant potential of ERG has been evaluated in several disease models [86,87]. The ability of ERG to localize and accumulate within mitochondria has been previously shown [88]. The therapeutic benefit of ERG is thought to be mediated by scavenging ROS (OH^●^, ONOO^−^), upregulation of antioxidant genes (NRF2, HSP70, SOD, GSH reductase and catalase) and downregulation of the pro-oxidant genes (NADPH oxidase). As an antioxidant that can accumulate in the mitochondria, its potential as a therapeutic in PE has been recently evaluated in a RUPP rat model of preeclampsia by Williamson et al. [8]. The authors showed that oral administration of ERG (at a dose of 25 mg/kg per day, GD11–19) attenuated hypertension and rescued fetal growth restriction in the RUPP rats. In addition, ERG treatment reduced circulating sFlt1 levels and mitochondrial H_2_O_2_ in kidneys of RUPP rats. Further, in a separate study published by the same group, ERG-treated RUPP rats showed higher levels of glutamylcysteine (metabolite associated with oxidative stress in mitochondria) and lower levels of metabolites associated with inflammation [89]. Collectively, findings from these two studies suggest that the antihypertensive and anti-inflammatory properties of ERG are mediated by preventing mitochondrial oxidative damage by its direct effects in the mitochondria. However, further studies exploring ERG beneficial effects on mitochondrial function in placenta and endothelial cells need to be conducted before ERG can be fully considered as a potential mitochondrial antioxidant for clinical use during pregnancy. The phase I study evaluating pharmacokinetics and safety of ERG in humans at doses of 5 or 25 mg daily administration showed rapid systemic absorption without safety concerns [90]. Further, another study evaluating chronic administration of ERG at doses of 400, 800, and 1600 mg per kg in Sprague Dawley rats did not cause any adverse effects [91]. Moreover, studies evaluating safety in pregnant rats did not show any adverse effects on the maternal pregnancy or fetus [92,93]. Interestingly, preeclampsia has been associated with increased erythrocyte ERG levels [94], which was thought to be due to increased SLC22A4 (ERG transporter) expression in response to inflammatory cytokines in PE as a protective mechanism to counteract oxidative stress. Overall, the existing data on the therapeutic potential of ERG is encouraging; however, further studies utilizing preeclamptic animal models are necessary to characterize mitochondrial antioxidant actions of ERG.

## 6. The Potential of Mitochondrial Antioxidants in Treating Preeclampsia

Although there have not been any clinical studies evaluating mitochondrial antioxidants in preeclamptic patients yet, the compelling preclinical data from multiple independent research groups are promising and certainly form a foundation for translational efforts of these molecules. Before considering any novel drug molecule to be a candidate for clinical evaluation in pregnancy, the impact of pregnancy-induced physiological changes and feto–placental transfer on the safety, pharmacokinetics, and efficacy should be demonstrated in animal models. In terms of an extensive characterization in animals and humans, MitoQ dominates all the other antioxidants discussed in this review (readers are encouraged to refer to the review on MitoQ by Smith et al. [74]). In recent years, MitoQ has been shown to be safe in at least in four independent studies involving pregnant rodents. However, it appears that administration of MitoQ during early administration exacerbates the preeclamptic symptoms in pregnant animals; hence, further evaluation on when to administer MitoQ during pregnancy and its safety profile must be understood before moving into clinical trials. Further, both MitoTempo and ERG have been reported to be safe in pregnant animals, and ours was the only study that evaluated MitoTempo in pregnant animals. Importantly, no signs of maternal or fetal toxicity were observed at the dose we used. While pharmacokinetics of ERG have been studied in one study, pharmacokinetics of MitoTempo are unavailable at this time. Thus, further studies are warranted for a thorough understanding of the safety and disposition of these molecules in pregnant animals. Given that no toxicity has been noted with any of these drugs in pregnant animal models, the potential of human placental transfer and fetal toxicity is assumed to be very low to none; however, further extensive evaluation using in vitro human placental models should be considered to validate these safety findings. It is worth noting that a few studies showing unchanged mitochondrial respiration in cytotrophoblasts and low mitochondrial H_2_O_2_ levels in PE placenta emphasize further thorough understanding of the source of oxidative stress and ROS metabolic regulation within the mitochondria of PE placenta. In addition, while a few studies reported no changes in respiration, others documented increased respiration rates in PE placental mitochondria with some of these studies noting differences between preterm vs. term placental bioenergetics (31–33). Such differences between preterm vs. term mitochondria are thought to exist due to differential expression of antioxidant defense systems (SOD, Catalase, GPRx) during the course of pregnancy. It is also critical to keep in mind that progression of preeclampsia pathogenesis is driven by a complex interplay of a variety of both placental and circulating factors. Based on the experimental data demonstrating a direct detrimental effect of such factors on oxidative phosphorylation and ROS production, the differential mitochondrial effects could also be driven by the dynamic expression of these factors during the disease progression. While these studies highlight placental mitochondrial adaptations in PE, we need more studies that aim to understand the mechanisms regulating placental mitochondrial function during pregnancy. Overall, the hypoxic milieu, PE pathological factors, and antioxidant systems may collectively dictate the extent of mitochondrial damage at a given time during the course of pregnancy. Moreover, it may be important to consider the race of certain patient populations and determine if there are genetic or regional differences among the races in how the placentas respond to pregnancy in the presence and absence of hypertensive disorders of pregnancy. Nevertheless, the literature thus far offers convincing evidence for existence of mitochondrial dysfunction during PE at the level of the placenta, which is then indirectly involved in mt dysfunction in the periphery. Thus we must be cautious about the potential of targeted antioxidants in treating this disease in pregnant women. Until further evaluation of additional factors such as racial differences or obesity are better investigated in pregnant women and how these factors may influence placental mitochondrial function, additional studies to decrease mt ROS may not be beneficial for certain pregnant populations.

## 7. Conclusions

The disappointing results from vitamin E and vitamin C clinical trials shifted the focus of PE research away from targeted antioxidant therapy in PE. However, it is widely known that delivering antioxidants directly to the source of oxidative stress could be an effective strategy to combat oxidative stress in several disease states. While several studies have shown mitochondrial oxidative stress within the preeclamptic placenta, two independent recent studies showed PE placental mitochondria produce low ROS (H_2_O_2_) in comparison to normal pregnant placental mitochondria [32,34]. While this finding may contradict our existing understanding of mitochondrial oxidative stress in PE, low H_2_O_2_ levels seen in PE placenta may have been simply a result of dysregulated ROS metabolic systems or the product measured, or could have been as simple as an underlying difference between races, thus warranting a further investigation of ROS dynamics in PE mitochondria. Importantly, we and others have shown targeting mitochondrial oxidative stress using mitochondrial-specific antioxidants such as MitoQ and MitoTempo would attenuate hypertension and improve fetal outcomes in animal models of PE. Other antioxidants such as ERG that are not required to be tagged with TPP (as have transporters for mitochondrial accumulation) have also shown promise in PE animal models. Further, the safety and efficacy findings of these antioxidant molecules are encouraging and certainly form a strong foundation for further characterization and evaluation in PE clinical trials. However, until we have a better understanding of extenuating factors (obesity and race) and how they affect placental mt function, we must proceed with caution and continue our preclinical studies before proceeding to utmost important clinical studies in the search for new therapeutics for an old disease.
